# Sequence2Script: A Web-Based Tool for Translation of Pharmacogenetic Data Into Evidence-Based Prescribing Recommendations

**DOI:** 10.3389/fphar.2021.636650

**Published:** 2021-03-18

**Authors:** Chad A. Bousman, Patrick Wu, Katherine J. Aitchison, Tony Cheng

**Affiliations:** ^1^ Departments of Medical Genetics, Psychiatry, and Physiology and Pharmacology, University of Calgary, Calgary, AB, Canada; ^2^ Alberta Children’s Hospital Research Institute, University of Calgary, Calgary, AB, Canada; ^3^ Mathison Centre for Mental Health Research & Education, Hotchkiss Brain Institute, Cumming School of Medicine, University of Calgary, Calgary, AB, Canada; ^4^ Cumming School of Medicine, University of Calgary, Calgary, AB, Canada; ^5^ Departments of Psychiatry, Medical Genetics and the Neuroscience and Mental Health Institute, University of Alberta, Edmonton, AB, Canada; ^6^ Ushiosoft Corporation, Calgary, AB, Canada

**Keywords:** pharmacogenenomics and personalized medicine, prescription drug, tool, pharmacogenetic, decision-making

## Abstract

Pharmacogenomic (PGx) testing has emerged as an effective strategy for informing drug selection and dosing. This has led to an increase in the use of PGx testing in the clinic and has catalyzed the emergence of a burgeoning commercial PGx testing industry. However, not all PGx tests are equivalent in their approach to translating testing results into prescribing recommendations, due to an absence of regulatory standards. As such, those generating and using PGx data require tools for ensuring the prescribing recommendations they are provided align with current peer-reviewed PGx-based prescribing guidelines developed by expert groups or approved product labels. Herein, we present Sequence2Script (sequence2script.com), a simple, free, and transparent web-based tool to assist in the efficient translation of PGx testing results into evidence-based prescribing recommendations. The tool was designed with a wide-range of user groups (e.g., healthcare providers, laboratory staff, researchers) in mind. The tool supports 97 gene-drug pairs with evidence-based prescribing guidelines, allows users to adjust recommendations for concomitant inhibitors and inducers, and generates a clinical report summarizing the patient’s genotype, inferred phenotype, phenoconverted phenotype (if applicable), and corresponding prescribing recommendations. In this paper, we describe each of the tool’s features, provide use case examples, and discuss limitations of and future development plans for the tool. Although we recognize that Sequecnce2Script may not meet the needs of every user, the hope is that this novel tool will facilitate more standardized use of PGx testing results and reduce barriers to implementing these results into practice.

## Introduction

Drug therapies are an essential component of healthcare delivery but people exhibit variable response to these therapies and unexpected outcomes are routine. To address this variability at the bedside, personalized prescribing strategies such pharmacogenomic (PGx) testing have been developed and implemented. PGx testing has been successfully implemented in medical centers and health systems across North America, Europe and Asia (Volpi et al., 2018; CPIC, 2019). However, most healthcare providers work in clinics, medical centers, or health systems that have not yet integrated PGx testing into their clinical workflows, resulting in a reliance on commercial laboratories for their PGx testing needs.

The last decade has witnessed a significant growth in commercial laboratories offering PGx testing and this growth is projected to continue over the next decade (Reports and Data, 2020). This growth however, has occurred in the absence of regulatory standards for reporting and interpreting PGx testing results, which in turn has created a situation where drug selection and dosing recommendations can differ depending on the laboratory performing the testing (Bousman and Dunlop, 2018). Simple and affordable remedies for this situation are not currently available. As such, when a healthcare provider orders a PGx test they have three options: 1) consider the recommendations as presented to them, 2) disregard the recommendations, or 3) manually cross-check each recommendation to ensure they align with peer-reviewed PGx-based prescribing guidelines developed by expert groups, such as the Clinical Pharmacogenetics Implementation Consortium (CPIC) (Relling and Klein, 2011) and Dutch Pharmacogenetics Working Group (DPWG) (Swen et al., 2011) or product labels approved by regulatory agencies (e.g., US Food & Drug Administration, FDA). The third option is ideal but it is neither feasible nor sustainable for busy healthcare providers to verify recommendations for accuracy and as such an efficient method for performing this task would be of clinical value.

In addition, most commercial PGx testing laboratories do not account for the potential impact that concomitant drugs can have on the genotype to phenotype translation process. This process assumes no concomitant drugs are present and uses an individual’s genotype (e.g., CYP2D6 *1/*2) to infer their phenotype (in this example, CYP2D6 normal metabolizer) (Caudle et al., 2020). In real-world clinical practice however, the concurrent use of multiple drugs is routine and can lead to a discordance between the genotype-inferred phenotype and the clinically observed phenotype. These so called phenoconversion events are common and are often attributed to the presence of potent inhibitors or inducers of a cytochrome (CYP) P450 enzyme (Preskorn et al., 2013; Klomp et al., 2020). For example, a patient genotyped as a CYP2D6 normal metabolizer who is taking paroxetine (a strong CYP2D6 inhibitor) will phenotypically resemble (phenoconvert to) a CYP2D6 poor metabolizer. Unless the phenconversion event is reversed by discontinuation of the concomitant inhibitor/inducer, phenoconversion can lead to the implementation of drug selection or dosing recommendations unsuitable for the patient and increase the risk of unwanted outcomes.

To address these challenges in PGx testing interpretation and implementation, we have developed a simple, free, and transparent web-based tool (Sequence2Script, sequence2script.com) to assist healthcare providers and other users of PGx data in the efficient translation of PGx testing results into evidence-based prescribing recommendations.

The development of Sequence2Script was initially a response to an unmet need among laboratory staff in Alberta, Canada, who required an efficient method for translating PGx testing results into evidence-based prescribing recommendations. However, during the development and testing of the tool, it became clear that this unmet need extended beyond laboratory staff. Conversations with healthcare providers, researchers and experts in the PGx community suggested that a tool to assist with the translation of PGx information into drug selection and dosing recommendations would be valuable to all of them. As a result, Sequence2Script was designed with a wide-range of potential user groups in mind. Herein, we describe the core features of this tool, provide use case examples, and discuss limitations and future development of the tool.

## Methods

### Supported Gene and Drug Content

At the time of writing, Sequence2Script supported 11 genes and 87 drugs associated with PGx prescribing guidelines developed by CPIC (Relling and Klein 2011), DPWG (Swen et al., 2011) and the FDA (Food and Drug Administration, 2017), representing a total of 97 gene-drug pairs (Table 1).

**TABLE 1:** Gene and drugs with pharmacogenomic-based prescribing guidelines that are supported by Sequence2Script.

Gene	Drugs
CYP2B6	Efavirenz
CYP2C19	Amitriptyline, citalopram, clobazam, clomipramine, clopidogrel, doxepin, escitalopram, esomeprazole, imipramine, lansoprazole, omeprazole, pantoprazole, prasugrel, sertraline, ticagrelor, trimipramine, voriconazole
CYP2C9	Aspirin, celecoxib, flurbiprofen, ibuprofen, lornoxicam, meloxicam, naproxen, phenytoin, piroxicam, tenoxicam, warfarin
CYP2D6	Amiodarone, amitriptyline, amphetamine, aripiprazole, atenolol, atomoxetine, bisoprolol, brexpiprazole, cavedilol, clomipramine, clonidine, clozapine, codeine, desipramine, doxepin, duloxetine, eliglustat, flecainide, fluoxetine, fluphenazine, fluvoxamine, haloperidol, hydrocodone, iloperidone, imipramine, methylphenidate, metoprolol, mirtazapine, moclobemide, nebivolol, nortriptyline, odansetron, olanzapine, oxycodone, paroxetine, perphenazine, pimozide, propafenone, propranolol, quetiapine, risperidone, tamoxifen, tetrabenazine, tramadol, tropisetron, trimipramine, venlafaxine, vortioxetine, zuclopenthixol
CYP3A5	Tacrolimus
HLA-A	Carbamazepine (*31:01)
HLA-B	Abacavir (*57:01), allopurinol (*58:01), carbamazepine (*15:02), oxcarbazepine (*15:02), phenytoin (*15:02)
NUDT15	Azathioprine, mercaptopurine, thioguanine
SLCO1B1	Atorvastatin, fluvastatin, rosuvastatin, simvastatin
TPMT	Azathioprine, mercaptopurine, thioguanine
VKORC1	Acenocoumarol, warfarin

### Algorithm and Data Resources

**FIGURE 1:**
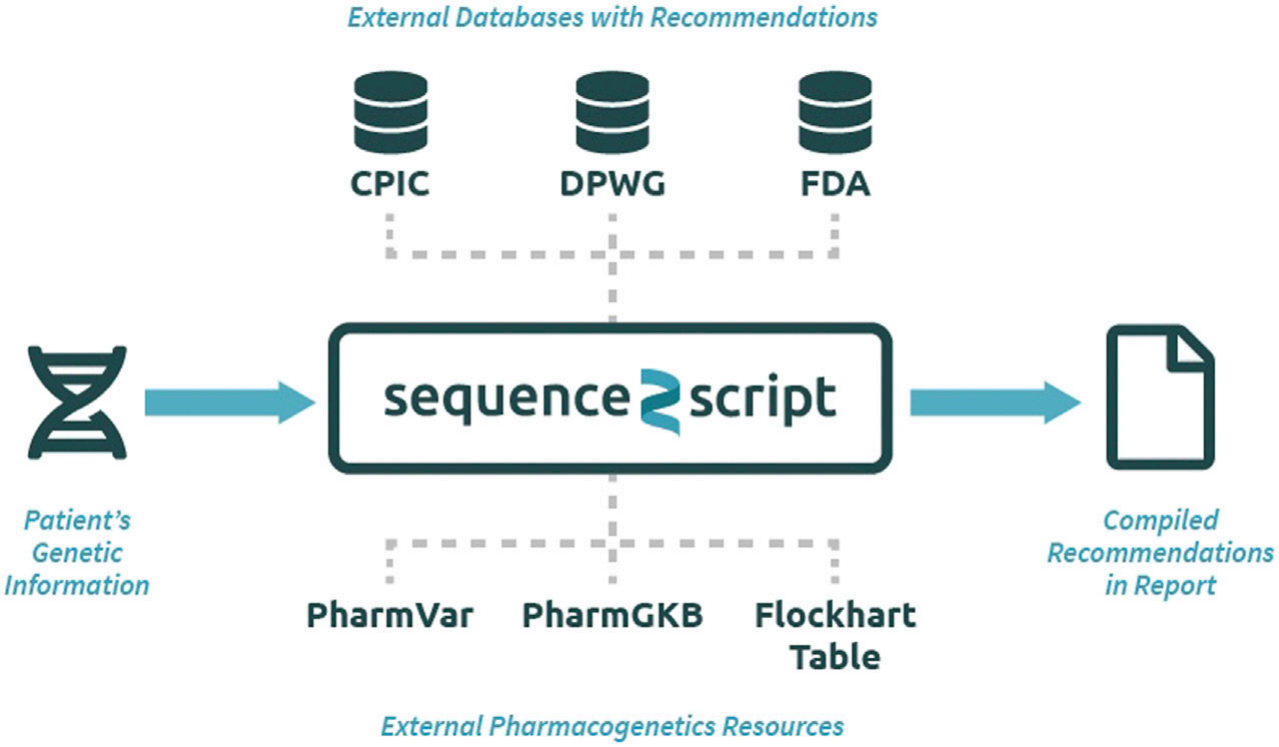
Overview of the Sequence2Script tool (sequence2script.com). CPIC, Clinical Pharmacogenetics Implementation Consortium; DPWG, Dutch Pharmacogenetics Working Group; FDA, Food & Drug Administration; PharmGKB, Pharmacogenomics Knowledgebase; PharmVar, Pharmcogene Variation Consortium.

The Sequence2Script tool does not use a proprietary algorithm to generate recommendations but instead integrates evidence-based PGx information from reputable sources into a single location for easy and efficient queries (Figure 1). All the recommendations produced by the tool can be traced back to their original source and reproduced manually. The data resources utilized by the tool include PGx prescribing guidelines created by CPIC (Relling and Klein, 2011), DPWG (Swen et al., 2011) and the FDA (Food and Drug Administration, 2017) as well as PGx information contained in the Pharmacogenomics Knowledgebase (PharmGKB) (Whirl-Carrillo et al., 2012), and Pharmacogene Variation Consortium (PharmVar) (Gaedigk et al., 2018). CYP450 substrate, inhibitor and inducer information are extracted from the Flockhart Table, a catalog of drug-drug interactions that are mediated by CYP450 enzymes (Flockhart, 2007). Sequence2Script does not directly interact with these data resources. Data from each source are manually extracted and curated by the lead author and his laboratory staff. The database is immediately updated when new CPIC and DPWG guidelines are published. The entire database is reviewed and updated annually. The date of the most recent database update is provided in the top right corner of every report produced by the tool.

### User Interface

**FIGURE 2:**
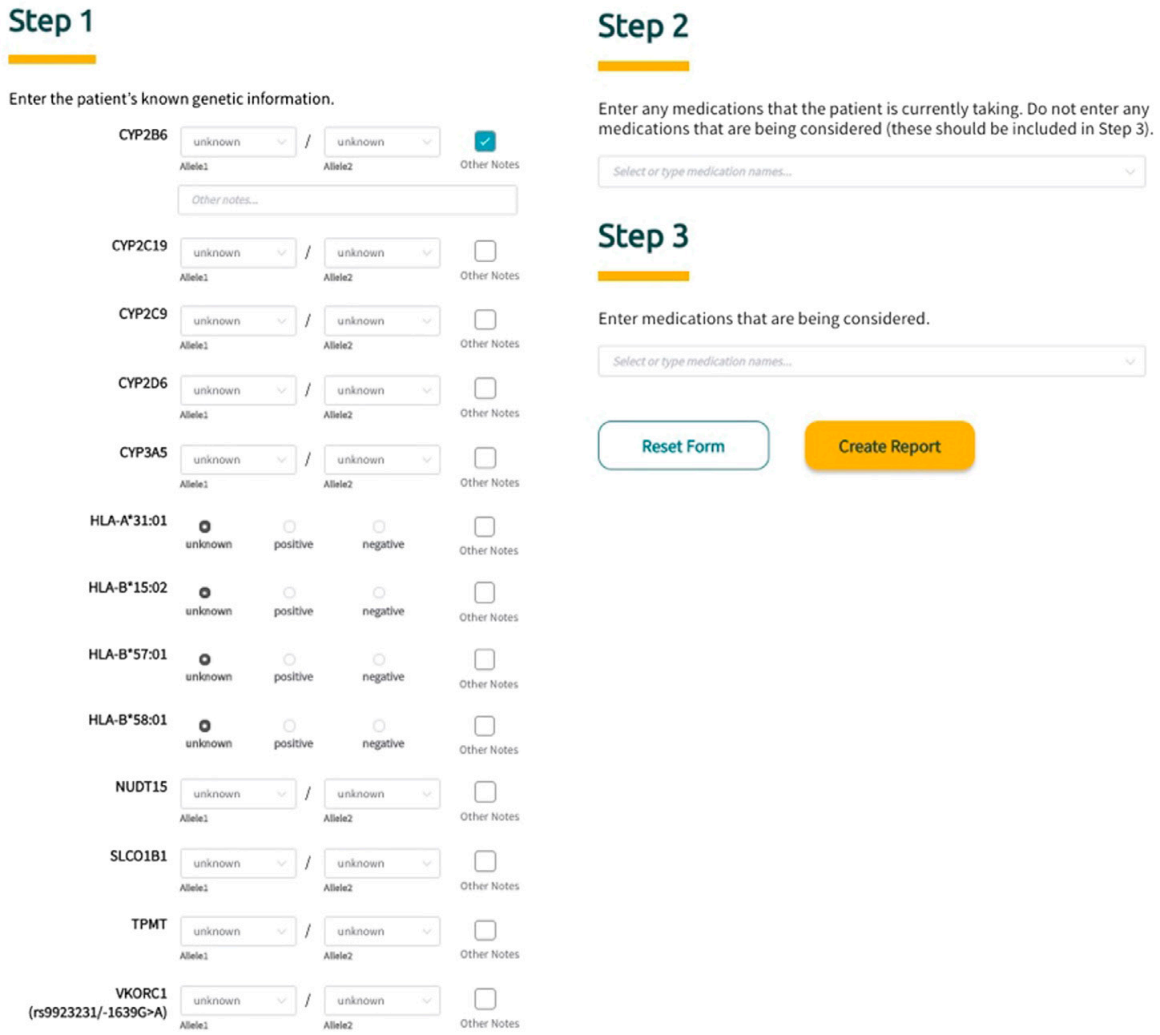
Screenshot of Sequence2Script’s three-step data input workflow.

Sequence2Script was developed and optimized for use with Google’s Chrome web browser. To fully utilize the core features of the tool (described in detail below), users are asked to input information in three steps (Figure 2). In Step 1, PGx information is entered into the tool using a combination of drop-down menus and radio buttons. The tool does not require data to be entered for all genes to generate a report. For each gene, the user has the option to include notes (free text) related to the PGx data being entered and these notes will appear in the genetic results section of the final report next to the corresponding gene. In Step 2, the user is given the option to enter current drugs that the patient is taking, which are then used to determine if phenoconversion adjustments (described in detail below) are required. Finally, in Step 3, the user has the option to enter drugs that are being considered for future use. Using this option extracts the recommendations (if available) associated with the specified drugs from the full list of recommendations and puts them in a separate box (Medications Being Considered) to facilitate quicker access to information that is most relevant to the user. Notably, none of the information entered into the tool by the user is saved and is not retrievable once the application is closed or refreshed.

### Core Features

The core features of the Sequence2Script tool include: 1) genotype to phenotype translation, 2) phenotype adjustments for concomitant drugs (i.e., phenoconversion adjustments), and 3) generation of drug selection and dosing recommendations. The tool does not calculate haplotypes or star (*) alleles from raw genotyping data. It exerts no quality control on user supplied genetic information and assumes the methods used by the user to derive haplotypes were aligned with current standards developed by expert groups such as PharmVar (Gaedigk et al., 2018).

### Genotype to Phenotype Translation

User supplied genotypes for the 11 supported genes are translated to phenotypes using gene-specific diplotype to phenotype tables maintained by PharmGKB (PharmGKB, 2020). These tables map each diplotype (e.g., CYP2C19 *1/*2) to an inferred phenotype (e.g., *CYP2C19* intermediate metabolizer) based on the current evidence derived from PharmVar (Gaedigk et al., 2018) and CPIC guidelines (Relling and Klein 2011). When applicable, consensus standardized terms for phenotypes are used (Caudle et al., 2017). If a user supplies a diplotype that cannot be mapped to an inferred phenotype an “indeterminate” phenotype result is displayed in the final report. If the user does not supply diplotype or genotype data for a particular gene an “unknown” phenotype will be displayed in the final report.

### Phenoconversion Adjustments

**FIGURE 3:**
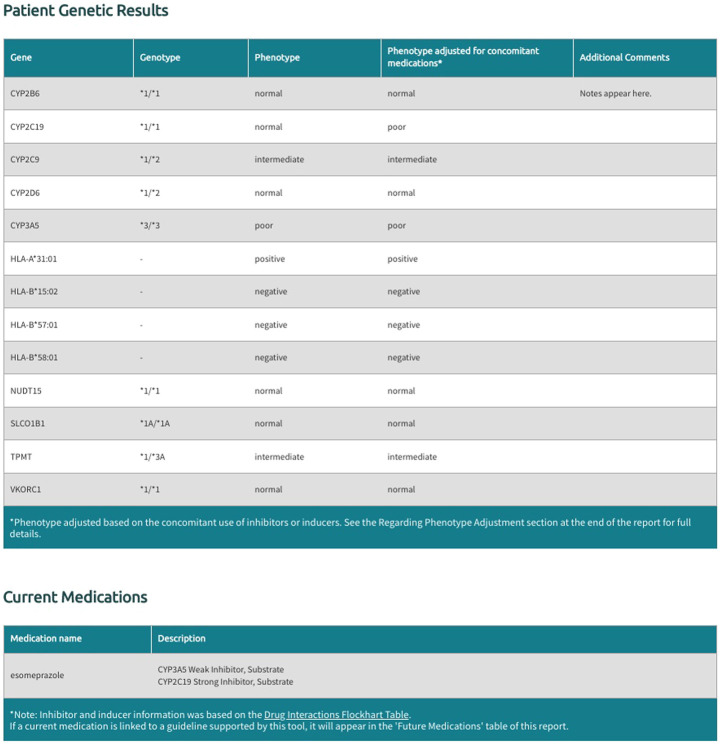
Example of genotype and phenotype report generated by Sequence2Script.

The Sequence2Script tool includes an option for users to account for concomitant drugs with the propensity to cause phenoconversion for the five supported CYP450 genes (*CYP2B6, CYP2C9, CYP2C19, CYP2D6, CYP3A5*). These phenoconverting drugs are currently derived from the Flockhart Table (Flockhart 2007) and are categorized as substrates, inhibitors, or inducers for each of the supported CYP450 genes. Inhibitors are further categorized as weak, moderate, or strong according to the fold increase in the plasma area under the curve (weak: 1.25 - 2-fold, moderate: >2-fold, strong: >5-fold) or percent decrease in clearance (weak: 20–49%, moderate: 50–80%, strong: >80%) of a substrate drug in the presence of the inhibitor (Flockhart 2007). Inducers are not categorized by strength as this information is not currently available in the Flockhart Table (Flockhart 2007). Sequence2Script only adjusts CYP450 phenotypes in the presence of a moderate inhibitor, strong inhibitor or inducer but displays all user supplied concomitant inhibitors and substrates in the final report (Figure 3).

There is currently no consensus on a method for adjusting inferred phenotypes when concomitant inhibitors or inducers are present. Sequence2Script uses a simple adjustment strategy. If the tool user indicates a moderate inhibitor is present, the inferred phenotype is converted to the next lower activity phenotype (e.g., a normal metabolizer is converted to an intermediate metabolizer), whereas in the presence of a strong inhibitor the inferred phenotype is converted to a poor metabolizer, regardless of the inferred phenotype. In the presence of an inducer the inferred phenotype is converted to the next higher activity phenotype (e.g., an intermediate metabolizer is converted to a normal metabolizer). The tool does not perform adjustments for poor metabolizers in the presence of inhibitors or ultrarapid metabolizers in the presence of inducers because these phenotypes already represent the two extremes of the phenotype continuum. In cases where both a concomitant inhibitor and an inducer are supplied by the tool user, the inferred phenotype in question is not converted as the evidence needed to guide the conversion in these situations is limited.

### Generation of Recommendations

Inferred or phenoconverted phenotypes for each of the 11 supported genes are cross-referenced with drug selection and dosing recommendations based on published guidelines (i.e., CPIC and DPWG) and FDA product labels. When a gene-drug pair has recommendations from more than one of these sources, Sequence2Script selects a recommendation to display in the final report based on a preference hierarchy. CPIC recommendations are given preference over DPWG and FDA recommendations, while DPWG recommendations are preferred over FDA recommendations. The preference hierarchy is not necessary a reflection of the recommendation’s validity but rather transparency and accessibility. CPIC’s guideline development process (Caudle et al., 2014) is the most transparent of the three sources and their guidelines are highly accessible and annotated.

**FIGURE 4:**
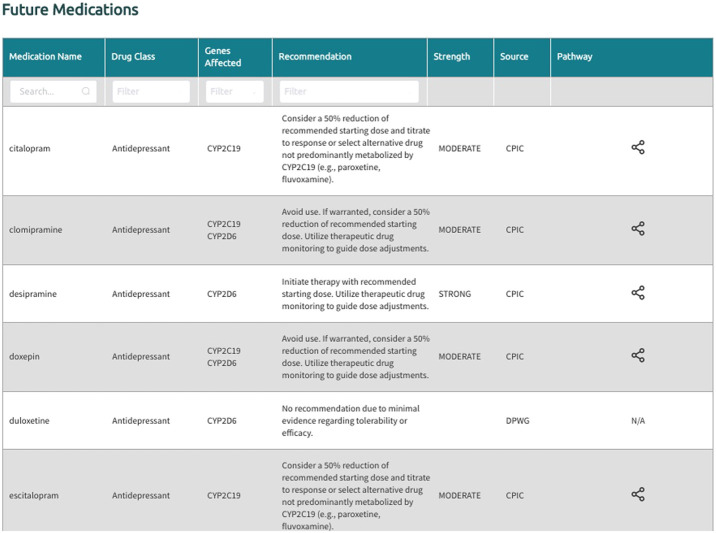
Excerpt of the ‘Future Medications’ section generated by Sequence2Script.

**FIGURE 5:**
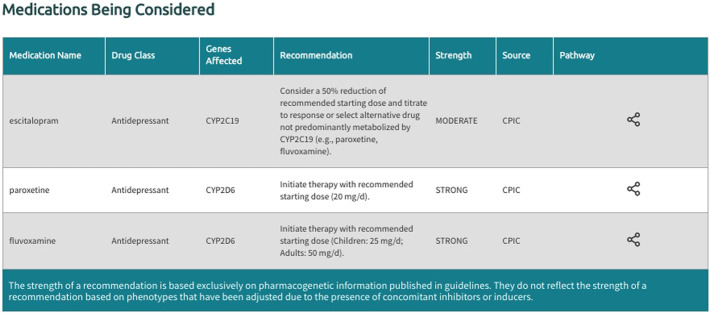
Excerpt of the ‘Medications being Considered’ section generated by Sequence2Script.

In the generated final report, recommendations are displayed in a uniform fashion for all supported gene-drug pairs and can be filtered by drug, drug class, gene, and recommendation type (i.e., drugs with or without an actionable recommendation) (Figure 4). If ‘medications being considered’ were provided in Step 3, those medications and corresponding recommendations will appear in a separate table in the final report (Figure 5). If phenoconversion adjustments were made, recommendations will reflect the phenoconverted phenotype rather than the inferred phenotype. When available, each recommendation is also accompanied by information on the strength and source of the recommendation as well as the PharmGKB gene-drug pathway. The strength of the recommendations are aligned with CPIC’s definitions for strong (high quality evidence, desirable effects clearly outweigh the undesirable effects), moderate (moderate quality evidence, desirable effects clearly outweigh the undesirable effects), optional (weak evidence or desirable effects are closely balanced with undesirable effects) or no recommendation (insufficient evidence, confidence, or agreement) (CPIC.). The source (CPIC, DPWG, or FDA) and PharmGKB pathway information displayed in the report are hyperlinked to allow efficient access to the original information, which is housed within the PharmGKB website.

### Customizable Features

As mention above, Sequence2Script was developed for a wide-range of user groups. However, during the development of the tool we recognized that some laboratories and healthcare centers or systems looking to start a PGx testing service may require customizable features to align with local laboratory reporting standards and practices. To address this anticipated need, Sequence2Script can be customized to include labeled reports with patient information and specimen information as well as a printable wallet card for patients to carry with them (Supplementary Figure S1). These customizable features are not included in the free version of the tool as they require consultation with our development and design team on a cost-recovery basis.

## Results

Our tool has potential utility for a wide-range of potential user groups. Here, we highlight three use case examples that involve clinical laboratories, healthcare providers, and researchers. These use case examples are not meant to be inclusive of all potential uses for the Sequence2Script tool nor are they meant to be generalizable to all settings.

### Clinical Laboratories

As previously stated, the catalyst for developing Sequence2Script was an identified unmet need for an efficient method for creating evidence-based PGx testing reports in a clinical laboratory setting. Although commercial options are available, these options are cost prohibitive for many laboratories, particularly in publicly funded healthcare settings. Thus, many clinical laboratory directors wanting to implement PGx testing must manually translate PGx information into clinical recommendations or develop their own in-house solution. The former option creates a bottleneck in the clinical workflow and can extend turnaround times, while the latter approach requires investment (time and money) and expertize that many laboratories may not have. Sequence2Script offers a free, efficient, and evidence-based solution to this problem and can be customized (as described above) to align with local laboratory reporting requirements.

### Healthcare Providers

PGx testing is now widely available and, in many cases, can be ordered by patients without the involvement of a healthcare provider (Bousman and Hopwood 2016; Haga and Kantor 2018). As a result, the probability that a healthcare provider will be presented with PGx testing results is expected to increase (Bousman et al., 2019). This situation can be problematic in that many healthcare providers do not feel comfortable or confident using these test results due, in part, to concerns about the validity of the recommendations (Veilleux et al., 2020) or lack of transparency in how the recommendations were derived (Bousman and Eyre, 2020). In these cases, Sequence2Script can serve as a second opinion. For example, PGx testing results obtained from a commercial PGx testing company can be entered into the tool and the recommendations can be compared to the current evidence-base, increasing confidence or highlighting discrepancies in the PGx recommendations provided to them. The tool also offers healthcare providers a mechanism for interpreting the PGx recommendations within the context of their patient’s current drug regimen by adjusting recommendations for concomitant inhibitors or inducers, a feature rarely offered by PGx testing providers or commercial PGx translation tools.

### PGx Research Community

The PGx research community is generally well-informed and capable of translating PGx information into clinical recommendations. However, as the number of gene-drug pairs associated with prescribing guidelines grows, even the most-well informed members of the PGx community will appreciate a quick and accessible tool that can efficiently perform this task. Furthermore, knowledge translation and transfer are crucial activities in PGx research but the tools required to facilitate these activities are often not available. With the growth of precision health and more specifically PGx research, we anticipate that tools such as Sequence2Script will assist researchers in meeting their knowledge translation and transfer objectives. For example, Sequence2Script provides opportunities for clinical researchers to share their results, when appropriate, with stakeholders (e.g., research participants) in a meaningful and personalized format.

## Discussion

The translation of PGx testing results into evidence-based recommendations is not a trivial process and simple tools to guide this process are often not available or accessible to providers or end users of PGx testing. Herein, we described Sequence2Script, a free web-based tool that was developed to facilitate the efficient translation of PGx testing results into evidence-based recommendations.

We anticipate a wide-range of user groups will utilize this tool and that the extent of its use will range from occasional to routine. Regardless of the user group or frequency of use, the hope is that Sequence2Script will facilitate more standardized use of PGx testing results and reduce barriers to implementing these results into practice. However, there are several notable limitations that remain to be addressed and should be considered when using Sequence2Script. First, not all gene-drug pairs with evidence-based prescribing recommendations are supported by the tool. Drugs associated with *DPYD* (capecitabine, fluorouracil, tegafur), *G6PD* (raburicase), *UGT1A1* (irinotecan, atazanavir) and *CACNA1S/RYR1* (potent volatile anesthetic agents, succinylcholine) are currently excluded but will be added in a future version of Sequence2Script. Second, the methods used to perform phenoconversion adjustments of inferred phenotypes are blunt and will need to be further refined as the evidence evolves. This will include expanding the number of inhibitors and inducers supported by the tool and capturing the nuances in the strength and specificity of these inhibitors and inducers to specific CYP450 enzymes and their drug substrates as well as accounting for inhibitors and inducers of other enzymes (e.g., *TPMT*) and transporters (e.g., *SLCO1B1*). Third, the tool does not currently support batched PGx data. Sequence2Script users are required to manually enter PGx data for each patient separately. An upload option capable of accepting PGx data in batches is a feature being developed for a future version of the tool. Fourth, the tool does not save data supplied or reports produced by the user. As such, updates to a specific report will require re-entry of data and re-generation of the report. Furthermore, the tool does not provide notifications to users when updates to the tool (e.g., new features, revised recommendations) have been made. However, at the top of every Sequence2Script report a generation date is included along with the date the database was last updated. Fifth, the translation of genotypes to phenotypes to recommendations are based on expert guidelines but the implementation of these guidelines by our tool has not undergone external validation. As such, utilization of the tool is at the users own risk. In addition, the information produced by the tool is intended to be interpreted by a licensed physician or other licensed healthcare professional, who has ultimate responsibility for all therapeutic decisions based on the individual characteristics of the patient, of the drugs prescribed and a comprehensive interpretation of the Sequence2Script report. Sequence2Script accepts no responsibility for any modification or redistribution of a generated report and is not liable for any actions taken by individuals based on the information provided, or for any inaccuracies, errors, or omissions in the information contained in the report. Finally, Sequence2Script reports are not currently designed for direct integration into electronic health records but in future versions the tool will have an option to generate the PGx report in a computer-readable format.

In conclusion, Sequence2Script is a free web-based tool to aid the translation of PGx data into evidence-based drug selection and dosing recommendations. The tool supports 97 gene-drug pairs, allows users to adjust recommendations for concomitant inhibitors and inducers, and generates a clinical report summarizing the patient’s genotype, inferred phenotype, phenoconverted phenotype (if applicable), and corresponding prescribing recommendations. To our knowledge, this is the first and only free tool with these features. However, the expectation is that our tool will evolve with the PGx knowledge and new features, discussed herein, will be introduced in future versions. We encourage users of the tool to provide feedback (S2Sfeedback@gmail.com) on their experiences and suggest features for further development. In collaboration with its users, we hope that Sequence2Script will become a valuable addition to the PGx implementation toolbox.

## Data Availability

The original contributions presented in the study are included in the article/Supplementary material, further inquiries can be directed to the corresponding author.
