# Supplementary material for: Sequence2Script: A Web-Based Tool for Translation of Pharmacogenetic Data Into Evidence-Based Prescribing Recommendations
Source: Front Pharmacol. 2021 Mar 18;12:636650. doi: 10.3389/fphar.2021.636650 (PMC8015939; doi:10.3389/fphar.2021.636650)
Supplement: Supplementary file 1 [file image1.pdf]

SUPPLEMENTARY MATERIAL

Sequence2Script: A web-based tool for translation of pharmacogenetic data into evidence-based prescribing recommendations

Figure S1. Example of a Sequence2Script Personal Pharmacogenomics Information Card

Pharmacogenetics Information

sequence2script

Gene	Genotype	Phenotype	Actionable medications *(modifications suggested)*
CYP2B6	*1 / *1	normal	-
CYP2C19	*1 / *1	normal	amitriptyline, clomipramine, doxepin, imipramine, trimipramine
CYP2C9	*1 / *2	intermediate	phenytoin, warfarin
CYP2D6	*1 / *5	intermediate	amitriptyline, atomoxetine, clomipramine, desipramine, doxepin, eliglustat, flecainide, imipramine, metoprolol, nortriptyline, pimozide, propafenone, tamoxifen, tramadol, trimipramine, venlafaxine, zuclopenthixol
CYP3A5	*3 / *3	poor	-
HLA-A*31-01	-	positive	carbamazepine
HLA-B*15:02	-	negative	carbamazepine, phenytoin
HLA-B*57:01	-	negative	-
HLA-B*58:01	-	negative	-
NUDT15	*1 / *1	normal	azathioprine, mercaptopurine, thioguanine
SLCO1B1	*1A / *1A	normal	-
TPMT	*1 / *3A	intermediate	azathioprine, mercaptopurine, thioguanine
VKORC1	*1 / *1	normal	warfarin

What is this card?

This card contains information that can help inform your healthcare providers about how your body processes or reacts to specific medications. Please keep this with you at all times.

<u>DO NOT</u> stop or change how you take your medications without speaking with a healthcare professional.

For use by healthcare professionals only

For additional clinical guidance on interpreting this report, the Calgary Clinical Pharmacology physician consultation service is available to assist you from **Monday–Friday**, from **9am–5pm** MST.

The Clinical Pharmacology physician can be reached by calling the Foothills Medical Centre switchboard at **403-944-1110** and asking to speak with the Clinical Pharmacology physician on call.

For more information, visit **sequence2script.com**

sequence2script

Personal Pharmacogenetics Information Card

For more information, visit: **sequence2script.com**

Patient Name: First Lastname
Date of Issue: Nov 20, 2020
